# Characterization and therapeutic evaluation of a novel phage S18-3 targeting poultry-derived *Enterobacter hormaechei* infection in mice

**DOI:** 10.1186/s12917-026-05451-6

**Published:** 2026-04-15

**Authors:** Rui Wang, Meiping Zhan, Quan Wang, Ningze Xia, Tianhao Chen, Shuang Gong, Qiong Zhang, Ling Liu, Yibo Zhang, Ruixia Zeng

**Affiliations:** 1 School of Basic Medical Sciences, Jinzhou Medical University, Jinzhou, Liaoning China; 2 The First School of Clinical Medicine, Jinzhou Medical University, Jinzhou, Liaoning China; 3 Department of Pathogen Biology, School of Basic Medical Sciences, Jinzhou Medical University, Jinzhou, Liaoning China; 4 Collaborative Innovation Center for Prevention and Control of Zoonoses, Jinzhou Medical University, Jinzhou, Liaoning China; 5 Department of Human Anatomy, School of Basic Medical Sciences, Jinzhou Medical University, Liaoning, China; 6 Liaoning Province Key Laboratory of Human Phenome Research, Jinzhou Medical University, Jinzhou, Liaoning China

**Keywords:** *Enterobacter hormaechei*, Bacteriophage therapy, Antibiotic resistance, Genomic analysis, Poultry infection

## Abstract

**Background:**

*Enterobacter hormaechei* (*E. hormaechei*) is an opportunistic pathogen in poultry, increasingly associated with multidrug resistance due to the extensive use of antibiotics in animal production. The emergence of resistant *E. hormaechei* strains poses a significant threat to poultry health and food safety, highlighting the urgent need for alternative antimicrobial strategies. This study aimed to isolate and characterize a novel lytic bacteriophage targeting *E. hormaechei* and to evaluate its biological properties and therapeutic potential in a gastrointestinal infection mice model.

**Results:**

A lytic phage, designated S18-3, was isolated from environmental water using *E. hormaechei* strain HZW1 isolated from diseased broiler liver. Phage S18-3 forms clear plaques (~ 0.6 cm) and exhibits an icosahedral head with a long tail. Optimal infectivity occurs at an MOI of 0.1. S18-3 remains stable from 30 to 50 °C and tolerates a broad pH range (pH 4–11). Whole-genome sequencing revealed no known virulence factors or antimicrobial resistance genes, indicating favorable genetic safety. In a murine intestinal infection model established by antibiotic pretreatment and oral challenge, *E. hormaechei* caused significant weight loss and mortality. Oral administration of S18-3 markedly improved survival and mitigated weight loss compared with infected controls. Phage S18-3 titers were detectable in feces in infection group, with only transient, low-level presence in blood. Histopathology showed no evident liver injury or inflammatory changes, suggesting no hepatotoxicity associated with phage treatment.

**Conclusions:**

Phage S18-3 is a stable, genetically safe lytic phage that effectively alleviates intestinal *E. hormaechei* infection in a murine model without observable adverse effects. These data support the potential of S18-3 as an antibiotic alternative for controlling *E. hormaechei* infections in poultry. Further validation in broiler models and farm-level settings is warranted to assess translational applicability in sustainable poultry production.

## Background

*Enterobacter hormaechei*, a member of the *Enterobacter cloacae* complex (ECC), has increasingly been isolated from livestock and poultry [1–4], clinical patients [5–7], and various environmental sources such as sewage [8, 9] and soil [10]. As a Gram-negative opportunistic pathogen, *E. hormaechei* has emerged as an important multidrug-resistant (MDR) bacterium in animal husbandry, associated with inflammation, bacteremia, and respiratory diseases in poultry and livestock [11–13]. The bacterium can spread through contaminated feed, water, and the environment, leading to cross-infection among flocks and potential zoonotic transmission via the food chain [14]. Moreover, isolates carrying resistance genes such as *qnrS*, *blaTEM-1*, and *mcr-9*, often co-harboring *blaNDM-1* and *blaKPC-2*, have been reported, conferring resistance to both carbapenems and colistin and severely limiting therapeutic options [15, 16].

Bacteriophages, viruses that specifically lyse bacteria, have gained renewed attention as promising alternatives to antibiotics in response to the global rise of antimicrobial resistance. Phages exhibit strong host specificity, can disrupt bacterial biofilms [17], and are considered safe for animals and the environment [18]. In poultry production, phage therapy has shown great potential for controlling bacterial pathogens. For example, oral or spray administration of phages has been shown to reduce *Salmonella* colonization in chicks [19] and block vertical transmission in breeder chickens [20], while broad-host-range phages have reduced *Campylobacter* contamination in poultry meat [21].

Given the increasing prevalence of MDR *E. hormaechei* and the lack of effective therapeutic strategies in poultry, this study aimed to isolate and characterize a novel lytic bacteriophage, S18-3, targeting avian-derived *E. hormaechei*. We further evaluated its biological properties, genomic safety, and in vivo therapeutic efficacy to assess its potential as a biocontrol agent against *E. hormaechei* infections in poultry.

## Methods

### Bacterial isolation and identification

The *E. hormaechei* strain HZW1 was isolated from liver tissues of five diseased broilers collected at a commercial farm in Tai’an County, Liaoning Province, China. Samples were streaked on Luria–Bertani (LB) agar (Beijing AoBoxing Bio-Tech Co., Ltd.) and incubated at 37 °C for 16–20 h. A single colony was selected for 16 S rRNA gene sequencing using primers 27 F (5′-AGAGTTTGATCMTGGCTCAG-3′) and 1492R (5′-GGTTACCTTGTTACGACTT-3′) (Sangon Biotech, China). Sequences were compared with NCBI BLAST and aligned in MEGA 11 to construct a neighbor-joining phylogenetic tree with 1,000 bootstrap replicates. The strain was stored in 25% glycerol at − 80 °C.

To evaluate the host range of phage S18-3, a collection of 13 additional *E. hormaechei* strains and 6 Escherichia coli strains were included. These strains were isolated from liver and fecal samples of chickens from the eight different poultry farms in Liaoning Province. All isolates were streaked on LB agar and incubated under the same conditions as HZW1. Species identification was performed using standard 16 S rRNA sequencing or previously confirmed by routine microbiological methods in the laboratory. Each isolate was cultured to exponential phase for use in phage susceptibility testing. *E. coli* ATCC 25,922 was used as the quality control strain.

### Antimicrobial susceptibility testing

Minimum inhibitory concentrations (MICs) were determined using the broth microdilution method following CLSI guidelines, with modifications (Kowalska-Krochmal & Dudek-Wicher, 2021; Tiwari et al., 2023). A bacterial suspension equivalent to 0.5 McFarland standard (approximately 1 × 10⁸ CFU/mL) was prepared and inoculated into a colorimetric microdilution system (Scenker Biological Technology Co., Ltd., China). Plates were incubated at 37 °C for 18–24 h.

### Phage isolation and characterization

Phages were isolated from seawater and river water samples collected in Jinzhou, Liaoning Province, China. Samples were filtered through 0.22 μm membranes to remove debris and bacterial cells, followed by enrichment with E. hormaechei HZW1 and incubation at 37 °C overnight. The enriched cultures were then centrifuged at 10,000×g for 10 min, and the supernatants were filtered through 0.22 μm membranes. Lytic phages were detected using the standard double-layer agar method. Individual plaques were picked and purified through three successive rounds of plaque isolation until uniform plaque morphology was obtained.

Phage lysates were subsequently subjected to endotoxin removal using Triton X-114 phase separation as commonly used in phage purification protocols. Briefly, Triton X-114 was added to a final concentration of 1% (v/v) and incubated at 4 °C for 30 min, followed by incubation at 37 °C for 10 min to induce phase separation and centrifugation at 12,000×g for 10 min. The aqueous phase was carefully collected, and the extraction was repeated 2–3 times. Residual Triton X-114 was removed by 3 kDa cutoff ultrafiltration, and purified phage preparations were stored stored in 25% glycerol at − 80 °C.

### Determination of multiplicity of infection and one-step growth curve

The optimal multiplicity of infection (MOI) was determined by infecting host bacteria with phage S18-3 at MOIs ranging from 10³ to 10⁻³, followed by incubation at 37 °C for 4 h. The MOI that yielded the highest phage titer was defined as the optimal MOI. Phage titers were quantified using the standard double-layer agar method and expressed as plaque-forming units per milliliter (PFU/mL) [22].

For the one-step growth curve [23],, host bacteria were infected with phage S18-3 at an MOI of 0.1 and allowed to adsorb for an appropriate period. The mixture was then centrifuged to remove unadsorbed phages, and the pellet was resuspended in fresh medium. The culture was incubated at 37 °C, and samples were collected at 10 min intervals over a period of 150 min to determine the latent period and burst size.

### pH and temperature stability

Phage suspensions (10⁹ PFU/mL) were incubated in buffers adjusted to pH values ranging from 2 to 13 at 37 °C for 1 h, or exposed to temperatures ranging from 30 to 80 °C for 1 h. The surviving phage titers were determined using the standard double-layer agar method and expressed as plaque-forming units per milliliter (PFU/mL). All experiments were performed in triplicate.

### Phage genome sequencing and analysis

Phage genomic DNA was extracted using a lambda phage DNA extraction kit (Leagene Biotechnology, China) according to the manufacturer’s instructions. Whole-genome sequencing was performed using the Illumina NovaSeq X Plus platform (Sinobiocore, China). Raw sequencing reads were assembled using MEGAHIT v1.2.9. Open reading frames (ORFs) were predicted and annotated using BLASTp searches against the Virulence Factor Database (VFDB), PHI-base v4.6, and the Comprehensive Antibiotic Resistance Database (CARD) to identify potential virulence and antibiotic resistance genes. Comparative genomic analysis was performed using BLASTn, and phylogenetic analysis was conducted using FastTree. Genome maps were visualized using Proksee.

### In vitro lytic activity

Log-phase bacterial suspensions (10⁸ CFU/mL) were mixed with phage S18-3 at multiplicities of infection (MOIs) of 0.01, 0.1, and 1 in 96-well plates. Bacterial growth was monitored by measuring the optical density at 600 nm (OD₆₀₀) at hourly intervals during incubation at 37 °C. LB medium without bacteria served as the blank control, and bacterial cultures without phage served as the negative control. All experiments were performed in triplicate.

### Animal experiments and safety evaluation

Twenty-four male C57BL/6J mice (18–22 g) were purchased from Changsheng Biotechnology Co., Ltd. (Animal Production License No. SCXK (Liao) 2020-0001). All mice were housed under controlled environmental conditions (temperature: 23 ± 1 °C; relative humidity: 50 ± 5%; 12-h light/dark cycle) with free access to water and standard chow. Before experiments, the mice were acclimatized for one week under standard laboratory conditions.

To facilitate intestinal colonization of *E. hormaechei*i, mice were pretreated with a broad-spectrum antibiotic cocktail administered via drinking water for 7 consecutive days, consisting of vancomycin (0.5 g/L), streptomycin (2 g/L), metronidazole (0.75 g/L), and fluconazole (0.5 g/L). After a 2-day washout period, mice were randomly divided into four groups (*n* = 6 per group): (1) PBS control group, (2) bacterial infection group, (3) phage treatment group, and(4) bacteria + phage treatment group.

Mice in the infection groups were orally gavaged daily with *E. hormaechei* HZW1 at a dose of 2 × 10⁹ CFU per mouse. Phage S18-3 was administered orally at a dose of 2 × 10⁸PFU per mouse, 1 h after bacterial challenge on day 1 only. Control mice received an equal volume of PBS.

Body weight was recorded daily throughout the experiment. Blood samples were collected daily from the tail vein to assess phage circulation. Fecal samples were collected daily to quantify phage titers. After 7 days, mice were euthanized, and liver tissues were harvested for histopathological examination using hematoxylin and eosin (H&E) staining.

### Statistical analysis

All experiments were performed in triplicate. Data are expressed as mean ± standard deviation (SD) and analyzed using GraphPad Prism 10.4.1. Statistical significance was determined by one-way ANOVA or Kaplan–Meier log-rank test. A p-value < 0.05 was considered statistically significant.

## Results

### Identification and antimicrobial profile of *Enterobacter hormaechei* HZW1

**Fig. 1:**
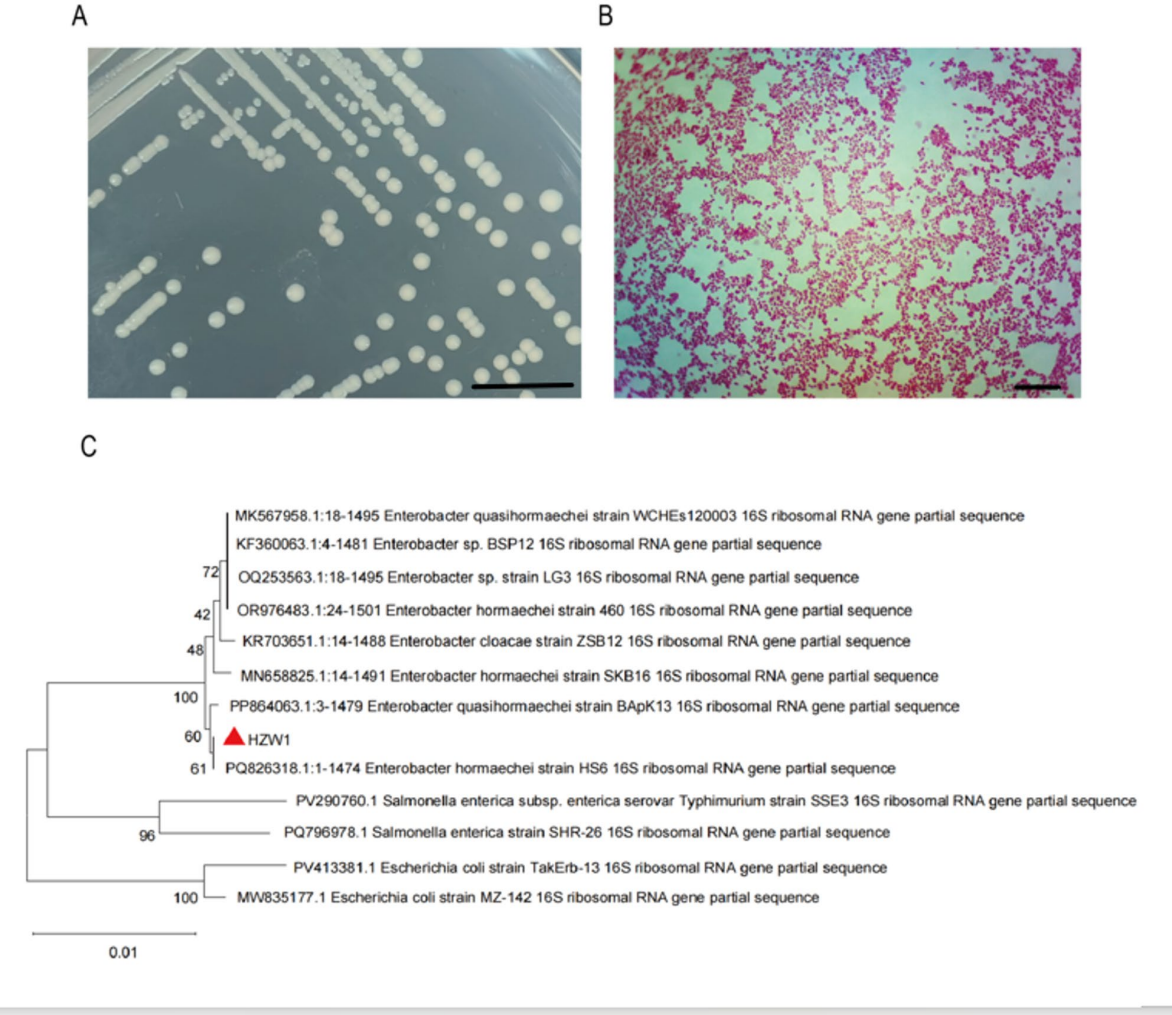
Isolation and Identification of *Enterobacter hormaechei* Strain HZW1. (**A**) Colonies of Enterobacter isolated from broiler chickens on LB agar. Scale bar = 10 mm. (**B**) Gram staining of Enterobacter under 1000× oil immersion microscopy. Scale bar = 10 μm. (**C**) Phylogenetic tree constructed based on the 16 S rRNA gene sequence of strain HZW1 using the neighbor-joining method with 1,000 bootstrap replicates

Colonies of the isolated strain appeared milky white, round, and smooth on LB agar after overnight incubation, consistent with the morphology of *Enterobacter* species (Fig. 1A). Gram staining revealed short, red-stained rods arranged singly or in short chains, confirming its Gram-negative nature (Fig. 1B).

Phylogenetic analysis based on the 16 S rRNA gene showed 100% sequence identity with *E. hormaechei* strain HS6, clustering in the same clade with 61% bootstrap support (Fig. 1C). The isolate was therefore designated *E. hormaechei* HZW1.

Antimicrobial susceptibility testing demonstrated resistance to cefazolin (MIC = 16 µg/mL) (Table 1), indicating resistant characteristics consistent with previous reports of *E. hormaechei* from poultry sources [1, 2].

**Table 1:** Antibiotic resistance profile of Enterobacter hormaechei S18-3

Antibiotic	MIC Value (μg/ml)	Interpretation
Ampicillin	≤8	S
Ampicillin/Sulbactam	≤8/4	S
Cefuroxime	≤8	S
Levofloxacin	≤0.12	S
Cefazolin	16	R
Cefoxitin	16	I
Cefepime	≤0.12	S
Amikacin	≤4	S
Gentamicin	≤1	S
Ceftazidime/Avibactam	≤0.5/4	S
Aztreonam	≤0.25	S
Colistin	≤2	I
Ciprofloxacin	≤0.015	S
Minocycline	≤1	S
Nitrofurantoin	≤16	S
Trimethoprim/Sulfamethoxazole	≤0.5/9.5	S
Cefoperazone/Sulbactam	≤0.5/0.2	S
Piperacillin/Tazobactam	≤4/4	S
Tigecycline	≤0.25	S
Ertapenem	≤0.015	S
Meropenem	≤0.06	S
Cefotaxime	≤0.12	S
Imipenem	≤0.5	S
Amoxicillin/Clavulanic Acid	16/8	I
Ceftazidime	≤0.5	S

*I* intermediate, *R* resistant, *S* susceptible

### Isolation and morphology of phage S18-3

**Fig. 2:**
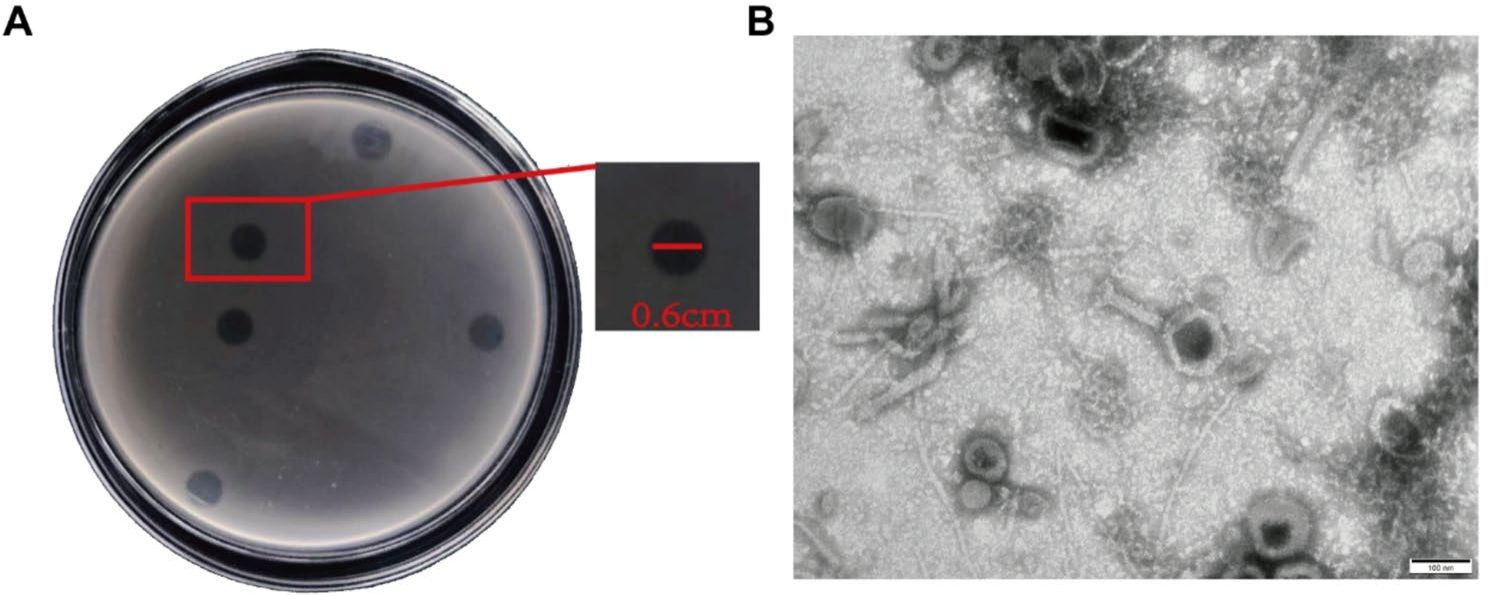
Morphological characteristics of phage S18-3. (**A**) Plaques formed by phage S18-3 on a double-layer agar plate. (**B**) Transmission electron micrograph of phage S18-3. Scale bar = 100 nm

A lytic phage infecting *E. hormaechei* HZW1, designated S18-3, was isolated from poultry farm sewage in Jinzhou, China. On double-layer agar plates, S18-3 produced clear, circular plaques approximately 0.6 cm in diameter (Fig. 2A). Transmission electron microscopy showed an icosahedral head and a distinct tail structure typical of the *Autographiviridae* family.(Fig. 2B).

### Host range of Phage S18-3

The host range analysis showed that phage S18-3 exhibited lytic activity against multiple *E. hormaechei* isolates derived from poultry sources (Table 2). Among the tested *E. hormaechei* strains, phage S18-3 was able to infect 6 out of 14 isolates, including strains isolated from chicken liver and feces. In contrast, no lytic activity was observed against any of the tested *Escherichia col*i strains, including the reference strain *E. coli* ATCC 25,922 and poultry-derived clinical isolates. These results indicate that phage S18-3 possesses a relatively narrow host range with specificity toward *E. hormaechei*.

**Table 2:** Host range testing of phages S18-3

Species	strain	Origin from	Lytic activity
*E. hormaechei*	HZW1	Chicken liver(Tai’an)	**+**
*E. hormaechei*	S15	Chicken liver(Jinzhou)	**-**
*E. hormaechei*	S21−3−2	Chicken liver(Linghai)	**+**
*E. hormaechei*	S18−1	Chicken liver(Jinzhou)	**-**
*E. hormaechei*	S19	Chicken liver(Shenyang)	**+**
*E. hormaechei*	S20	Chicken liver(Dalian)	**-**
*E. hormaechei*	S12−2	Chicken liver(Anshan)	**+**
*E. hormaechei*	S11	Chicken liver(Tai’an)	**+**
*E. hormaechei*	S10	Chicken liver(Yixian)	**-**
*E. hormaechei*	S9	Chicken feces(Yixian)	**+**
*E. hormaechei*	S6	Chicken feces(Jinzhou)	**-**
*E. hormaechei*	S5	Chicken feces(Linhai)	**-**
*E. hormaechei*	S3	Chicken feces(Dalian)	**-**
*E. hormaechei*	S1	Chicken feces(Shenyang)	**-**
*E.coli*	ATCC25922		**-**
*E.coli*	D12−2	Chicken liver(Jinzhou)	**-**
*E.coli*	A9−1	Chicken liver(JInzhou)	**-**
*E.coli*	D2−2	Chicken liver(Taian)	**-**
*E.coli*	A6B5	Chicken liver(Linghai)	**-**
*E.coli*	D3	Chicken liver(Dalian)	**-**

Infectivity was determined by spot assay. “+” indicates visible lysis zones on bacterial lawns, whereas “–” indicates no detectable lysis

### Biological characteristics of phage S18-3

**Fig. 3:**
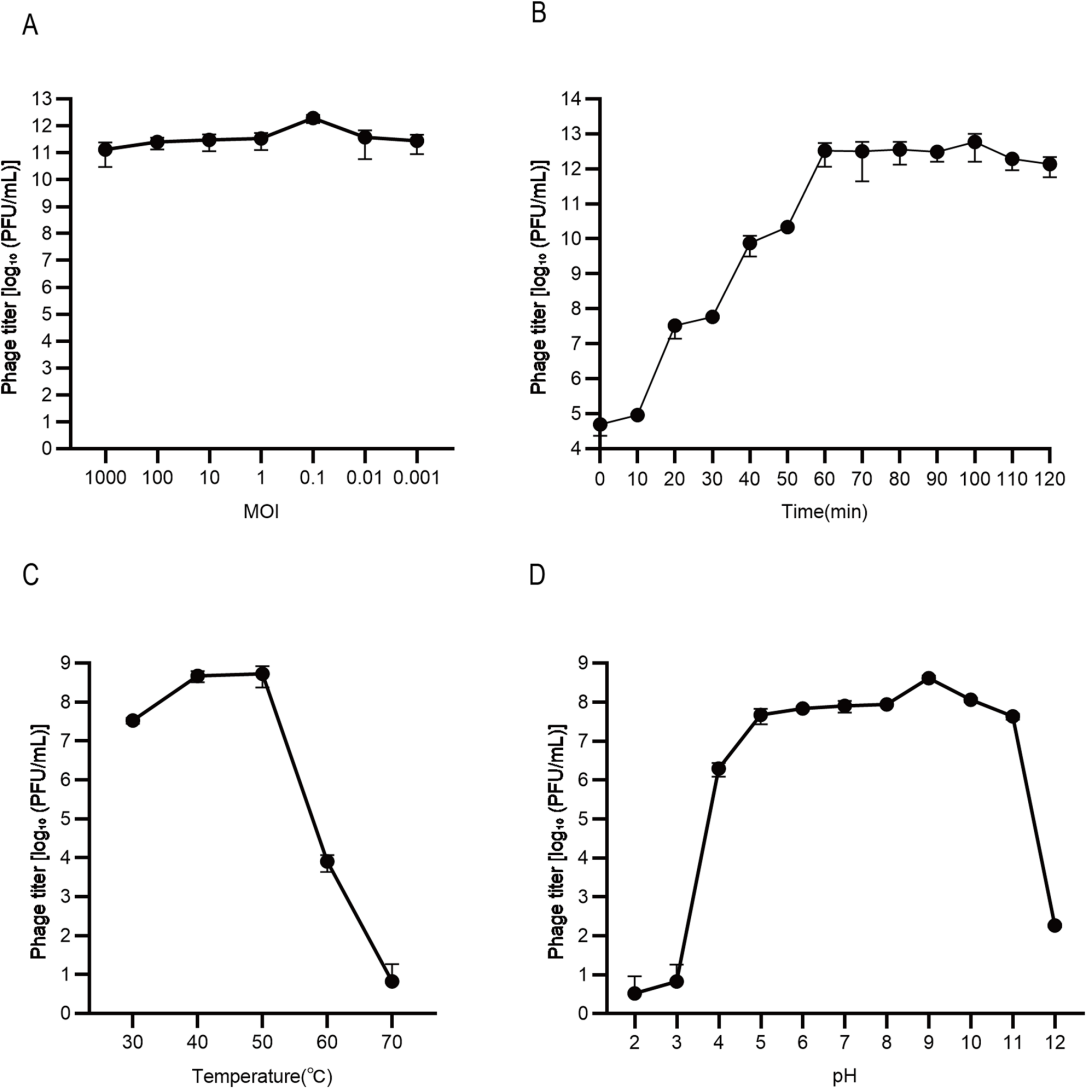
Biological characteristics of phage S18-3. **A** Optimal MOI assay; **B** One-step growth curve; **C** Thermal stability; **D** pH stability. All experiments were independently repeated three times

Phage S18-3 displayed the highest replication efficiency at an MOI of 0.1 (Fig. 3A). One-step growth curve analysis revealed a short latent period (~ 10 min), a lysis cycle of ~ 50 min, and a burst size of approximately 556 PFU per infected cell (Fig. 3B).

Thermal and pH stability assays showed that S18-3 remained stable at 30–50 °C and across pH 4–11 (Fig. 3C–D), demonstrating strong environmental tolerance suitable for biological applications in livestock production environments.

### Genomic characterization and phylogenetic analysis

**Fig. 4:**
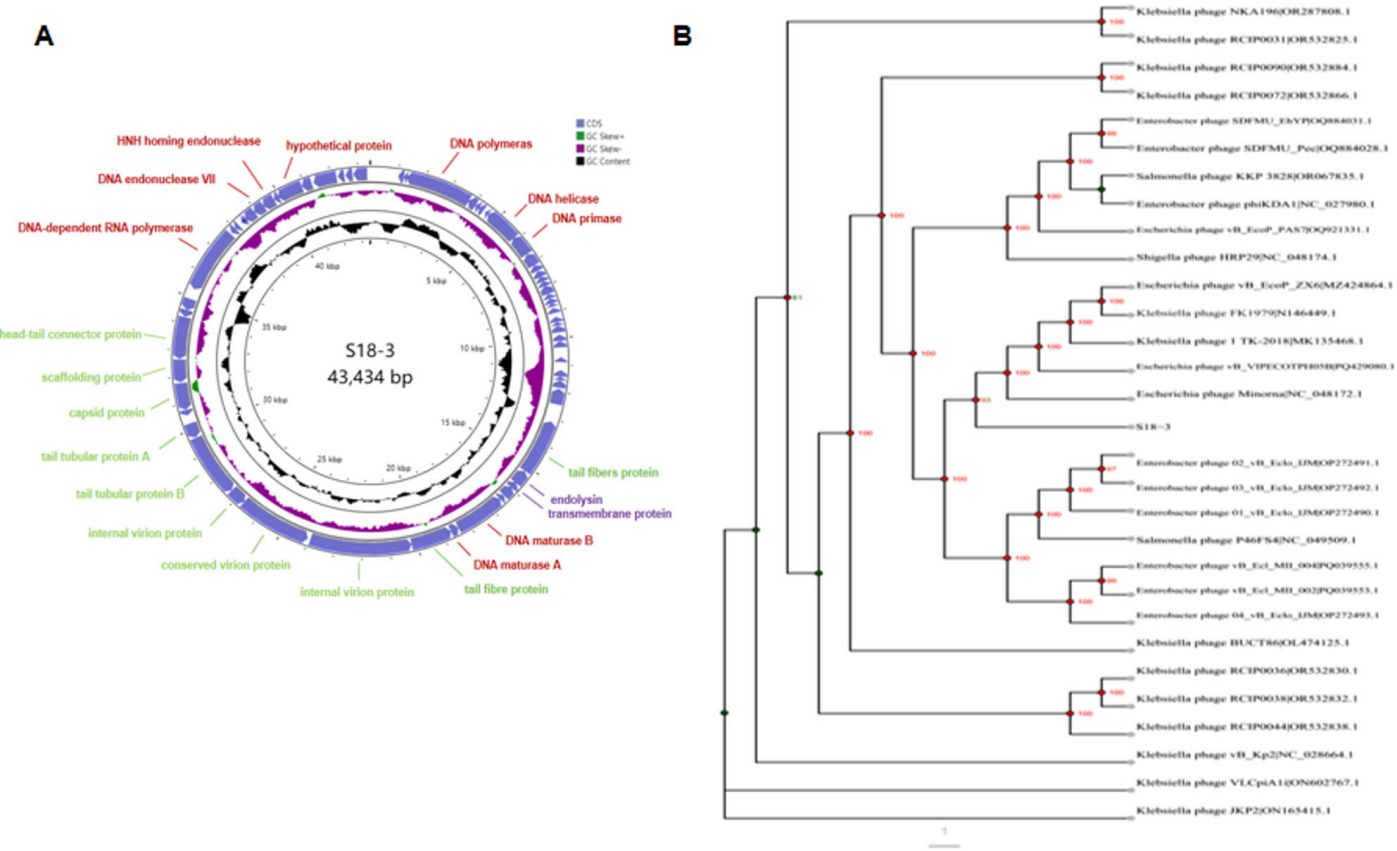
Genomic and phylogenetic features of S18-3. (**A**) Circular genome map of phage S18-3. Predicted coding sequences (CDSs) are shown in purple. GC content and GC skew are displayed in the inner rings. Key functional genes involved in replication, structural assembly, and host lysis are annotated.(**B**) Phylogenetic tree based on whole-genome sequences of S18-3 and related phages constructed using the neighbor-joining method. Bootstrap values are indicated at branch nodes

Whole-genome sequencing revealed that bacteriophage S18-3 possesses a linear double-stranded DNA genome of 43,434 bp with a GC content of 52.13% (Fig. 4A). A total of 63 open reading frames (ORFs) were predicted, all transcribed in the same direction. Functional annotation showed that these ORFs are organized into typical phage functional modules, including DNA replication and transcription (DNA polymerase, DNA helicase, primase, DNA ligase, and DNA-dependent RNA polymerase), structural proteins (capsid, scaffold, head–tail connector, tail tubular proteins, and tail fiber proteins), and host lysis-related proteins (endolysin and transmembrane protein). Several ORFs encoded hypothetical proteins with unknown functions.

Screening against the Virulence Factor Database (VFDB) and the Pathogen–Host Interactions database (PHI-base v4.6) revealed no known virulence-associated genes. In addition, no antibiotic resistance genes were identified using the Comprehensive Antibiotic Resistance Database (CARD), indicating that phage S18-3 is genetically safe for therapeutic application.

Phylogenetic analysis based on whole-genome sequences demonstrated that phage S18-3 clusters most closely with *Escherichia phage Minorna* (NC_048172.1), forming a well-supported clade distinct from other related phages (Fig. 4B). Although BLASTn analysis showed high nucleotide sequence similarity (approximately 93%) between S18-3 and several *Enterobacter* Enterobacter phages, including *phage 02_vB_Eclo_IJM* (OP272491.1) and *phage 03_vB_Eclo_IJM* (OP272492.1), these phages were positioned in adjacent but more distant branches in the phylogenetic tree.

Consistent with its phylogenetic placement, S18-3 was classified within the genus *Kayfunavirus* of the family *Autographiviridae*. Together, these results indicate that S18-3 shares a closer evolutionary relationship with *Escherichia phage Minorna* than with previously reported Enterobacter-infecting phages, highlighting the evolutionary conservation of this lineage across different *Enterobacteriaceae* hosts.

### In vitro antibacterial activity

**Fig. 5:**
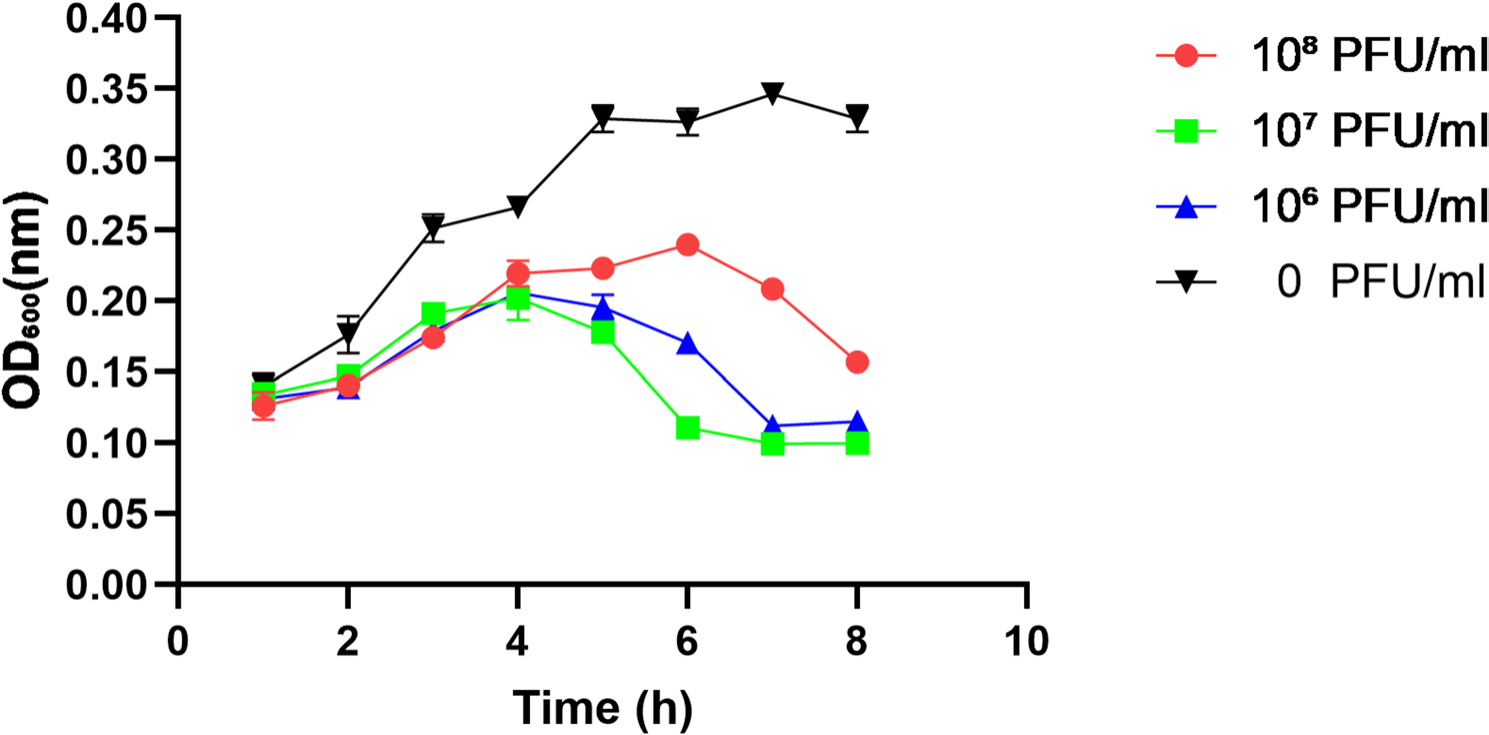
In vitro bacterial inhibition curves of S18-3 at different MOIs

Phage S18-3 demonstrated strong inhibitory activity against *E. hormaechei* HZW1 in vitro. At MOIs of 0.01, 0.1, and 1, bacterial optical density (OD₆₀₀) remained markedly lower than in the untreated control throughout the 8 h incubation period (Fig. 5), confirming efficient lysis and suppression of bacterial growth.

The growth dynamics of *E. hormaechei* were monitored by measuring optical density at 600 nm (OD600) over an 8-h incubation period following treatment with phage S18-3 at final concentrations of 10⁶, 10⁷, and 10⁸ PFU/mL. Bacteria without phage treatment (0 PFU/mL) served as the control. Phage treatment resulted in a concentration-dependent suppression of bacterial growth, with higher phage titers leading to earlier and more pronounced inhibition compared with the untreated control.

### In vivo antibacterial activity and safety evaluation

**Fig. 6:**
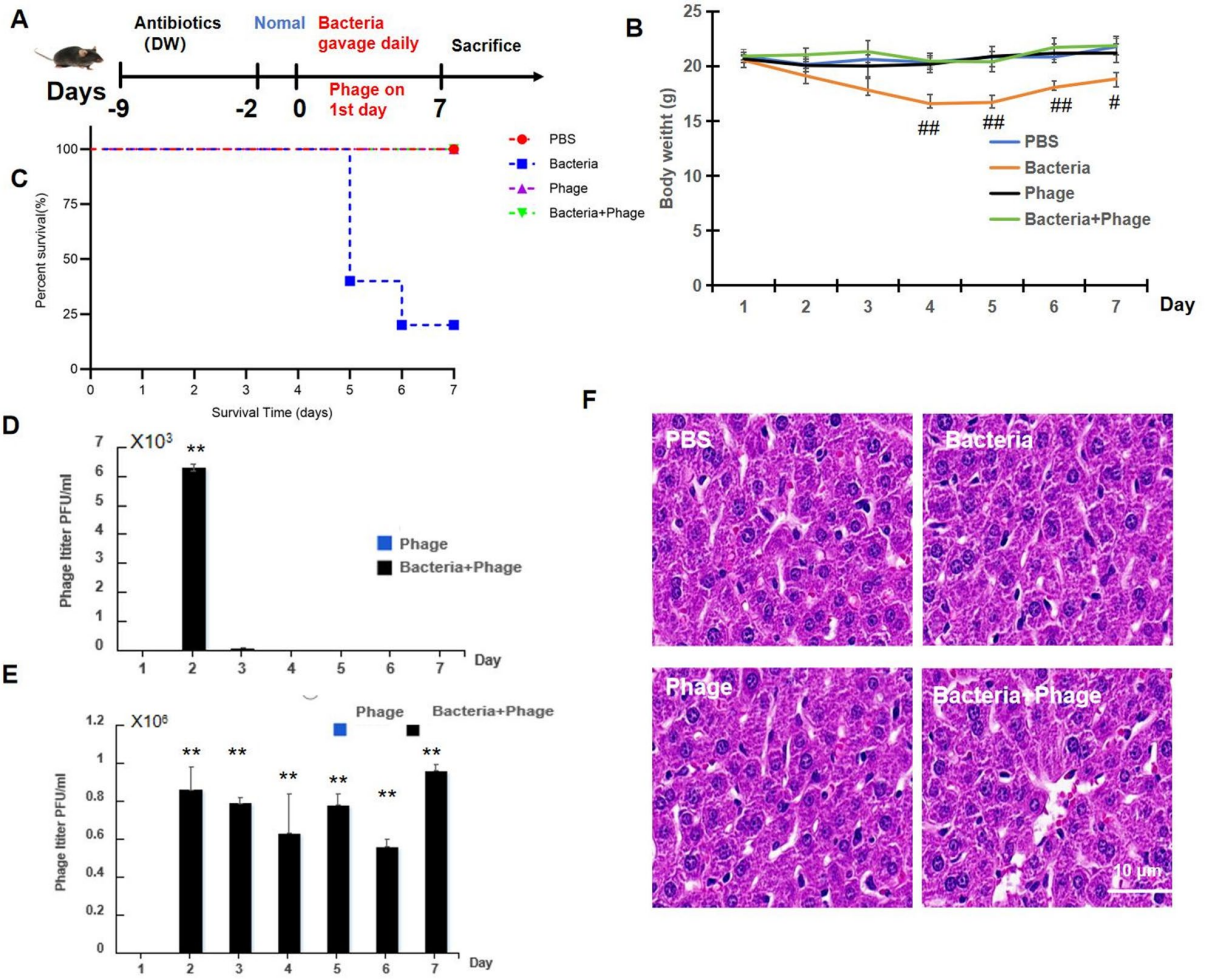
Oral phage therapy alleviates *E. hormaechei*–induced disease in mice. (**A**) Schematic illustration of the experimental design. Mice were pretreated with antibiotics, followed by oral bacterial challenge. Phage S18-3 was administered orally once, 1 h after bacterial gavage on day 1.(**B**) Changes in body weight of mice during the 7-day observation period (n = 6).(**C**) Survival curves of mice following bacterial infection and phage treatment (n = 6).(**D**) Phage titers detected in blood samples after oral administration (n = 3).(**E**) Phage titers detected in fecal samples over time (n = 3). (**F**) Representative hematoxylin and eosin (H&E)–stained liver sections from different treatment groups. Scale bar = 10 μm. Data are presented as mean ± SD. **p < 0.01 vs. PBS group; #*p* < 0.05, ##*p* < 0.01 vs. phage-treated group

To evaluate the therapeutic potential of phage S18-3 in vivo, a murine intestinal infection model was established, and the experimental design is shown in Fig. 6A. Mice were pretreated with antibiotics, followed by daily oral challenge with *E. hormaechei*. Phage S18-3 was administered once by oral gavage 1 h after bacterial infection on day 1.

As shown in Fig. 6B, mice in the bacterial infection group exhibited a significant and progressive loss of body weight compared with the PBS control group (##*p* < 0.01). In contrast, mice treated with phage S18-3 (bacteria + phage group) showed markedly attenuated weight loss and gradual recovery, with body weights significantly higher than those of the bacterial infection group (##*p* < 0.01). No significant changes were observed in the PBS or phage-only groups.

Survival analysis further demonstrated the protective effect of phage therapy (Fig. 6C). Mice infected with *E. hormaechei* showed substantial mortality, with survival dropping to approximately 20% by day 7. In contrast, all mice in the bacteria + phage group survived throughout the experimental period, indicating that phage S18-3 effectively prevented infection-associated lethality.

Phage dynamics in vivo were assessed by measuring phage titers in blood and fecal samples. As shown in Fig. 6D, phage particles were transiently detected in the bloodstream at low levels shortly after administration and rapidly declined thereafter, suggesting limited systemic circulation. In contrast, high levels of phage were consistently detected in fecal samples throughout the experiment (Fig. 6E), indicating successful colonization and persistence of S18-3 in the intestinal tract. Notably, phage titers in the bacteria + phage group were significantly higher than those in the phage-only group (**p < 0.01), suggesting active phage replication in the presence of the bacterial host.

Histopathological examination of liver tissues (Fig. 6F) revealed no obvious pathological alterations, including hepatocellular damage or inflammatory infiltration, in any of the experimental groups. The liver architecture remained intact across all groups, indicating that neither bacterial challenge under the current conditions nor phage treatment induced detectable hepatic injury.

Collectively, these results demonstrate that phage S18-3 confers significant protective effects against *E. hormaechei* infection in vivo, improving survival, alleviating weight loss, and maintaining tissue integrity, while exhibiting a favorable safety profile.

## Discussion

*Enterobacter hormaechei*, a member of the Enterobacter genus, is a Gram-negative opportunistic pathogen responsible for diverse infections in animals and humans, including bacteremia and sepsis [24]. Its increasing prevalence in livestock and poultry poses a serious challenge to animal health and agricultural productivity. The widespread misuse of antibiotics in animal farming has accelerated the dissemination of antibiotic resistance genes (ARGs), which can be propagated through contaminated soil, water, and feces [25]. Particularly concerning are the emergence of colistin-resistant [26] and carbapenem-resistant [27] *Enterobacteriaceae*, limiting the efficacy of conventional treatments and highlighting the urgent need for alternative antimicrobial strategies such as bacteriophage therapy.

In this study, a multidrug-resistant *E. hormaechei* strain, HZW1, was isolated from diseased broiler chickens, and a corresponding lytic phage, S18-3, was obtained and characterized. The isolation of such a phage from a poultry-associated environment emphasizes the ecological adaptation of phages to their bacterial hosts and their potential use in controlling resistant pathogens in the poultry industry. The resistance of strain HZW1 to cefazolin underscores the increasing difficulty of managing Enterobacter infections in livestock using antibiotics alone.

Phage S18-3 demonstrated typical lytic morphology and strong infectivity toward *E. hormaechei*. Its biological profile—short latent period (10 min), large burst size (556 PFU/cell), and optimal MOI of 0.1—indicates rapid replication and high lytic potential, comparable to phages previously reported against *Salmonella* [28] and *Escherichia coli* [29]. Stability across a broad range of pH (4–11) and temperature (30–50 °C) further supports its resilience under environmental and gastrointestinal conditions typical of poultry production systems. Such tolerance has also been observed in other heat- and acid-resistant phages used in poultry disease control [30].

Genomic analysis confirmed that S18-3 belongs to the *Kayfunavirus* genus within the *Autographiviridae* family. The absence of virulence and antibiotic resistance genes (based on VFDB, PHI-base v4.6, and CARD databases) suggests a favorable safety profile for therapeutic use. This finding aligns with previous reports of phages P.A-5 [8] and Ehp-YZU08/YZU10 [9], which also exhibited safe genomic characteristics, reinforcing the notion that Kayfunavirus-like phages are suitable candidates for biocontrol applications.The DNA replication and transcription module contained genes encoding key enzymes, including DNA polymerase, DNA helicase, DNA primase, and a DNA-dependent RNA polymerase. In addition, several nucleases, such as DNA endonuclease VII and an HNH homing endonuclease, were identified, suggesting active DNA processing during genome replication.Genes involved in DNA packaging were also identified, including putative DNA maturase A and B, corresponding to the terminase complex, as well as a head–tail connector protein. Structural protein genes encoding the capsid protein, scaffolding protein, tail tubular proteins, internal virion proteins, and tail fiber protein were clustered together, indicating a complete virion assembly module. Regarding host cell lysis, a putative endolysin and a putative transmembrane protein were identified in the late region of the genome, suggesting their involvement in bacterial cell wall degradation and membrane permeabilization during phage release. No canonical spanin (Rz/Rz-like) genes or other lysogeny-related genes, such as integrase or transcriptional repressors, were detected, supporting the strictly lytic lifestyle of phage S18-3.

In vivo, unlike systemic infection models based on intraperitoneal injection, *E. hormaechei* primarily colonizes the gastrointestinal tract and causes disease via intestinal translocation. Therefore, establishing an oral infection and treatment model more closely reflects the natural infection route and enhances the translational relevance of phage therapy. In this study, antibiotic pretreatment effectively facilitated intestinal colonization, enabling reproducible infection establishment. Oral administration of phage S18-3 significantly attenuated body weight loss, improved survival, and limited bacterial dissemination, while protecting liver tissue from secondary inflammatory damage (Figs. 6B–F). Notably, in vivo phage dynamics revealed that S18-3 was transiently detected in the bloodstream at low levels but persisted at high titers in fecal samples, indicating successful colonization and replication in the intestinal tract with minimal systemic circulation. Histopathological analysis further confirmed that liver architecture remained intact across all experimental groups, with no evidence of hepatocellular injury or inflammatory infiltration, supporting a favorable safety profile for S18-3. Similar protective effects of phage therapy have been documented in other animal models, where phage administration effectively reduced bacterial colonization and mortality [31]. Although this study employed a mouse model to evaluate efficacy, future validation in poultry models is warranted, given species-specific differences in immunity and gut microbiota. Nevertheless, previous research demonstrated that phage performance in murine models can correlate with outcomes in broilers, supporting the translational relevance of the current findings.

Despite these promising results, several challenges remain for the practical application of phage therapy in poultry farming. Phages typically exhibit a narrow host range, necessitating the formulation of phage cocktails to broaden antibacterial coverage. Studies have shown that oral administration of freeze-dried phage cocktails can reduce mortality by more than 60% in broiler infection models, with complete survival in treated groups [32]. Moreover, combined phage therapy not only prevents infection but also promotes weight gain and improved growth performance in infected broilers [33]. Beyond therapeutic use, phage application via spraying has been proven to lower *Salmonella* contamination in eggs, enhancing hatchability and chick quality [34]. These findings illustrate the diverse and practical potential of phages as biosecurity agents throughout poultry production chains.

## Conclusion

In this study, a novel lytic bacteriophage, S18-3, specific to *Enterobacter hormaechei* isolated from poultry, was identified and characterized. Phage S18-3 exhibited a short latent period, large burst size, and strong stability under a wide range of temperature and pH conditions. Whole-genome sequencing revealed no virulence or antibiotic resistance genes, confirming its genetic safety. In vivo experiments further demonstrated that S18-3 significantly improved the survival of infected mice without adverse effects, highlighting its therapeutic potential.

These findings indicate that S18-3 is a promising candidate for the biological control of *E. hormaechei* infections in poultry and may serve as an effective alternative to antibiotics in the context of rising antimicrobial resistance. Future studies should evaluate its efficacy in broiler models, explore synergistic effects with antibiotics, and assess its large-scale applicability in farm environments to support sustainable poultry production.

## Data Availability

The datasets generated and/or analyzed during the current study are available fromthe corresponding author on reasonable request.
